# Supplementary material for: Increased Susceptibility of Rousettus aegyptiacus Bats to Respiratory SARS-CoV-2 Challenge Despite Its Distinct Tropism for Gut Epithelia in Bats
Source: Viruses. 2024 Oct 31;16(11):1717. doi: 10.3390/v16111717 (PMC11598992; doi:10.3390/v16111717)
Supplement: Supplementary file 1 [file viruses-16-01717-s001.zip › viruses-3268034-supplementary.pdf]

Supplementary Data

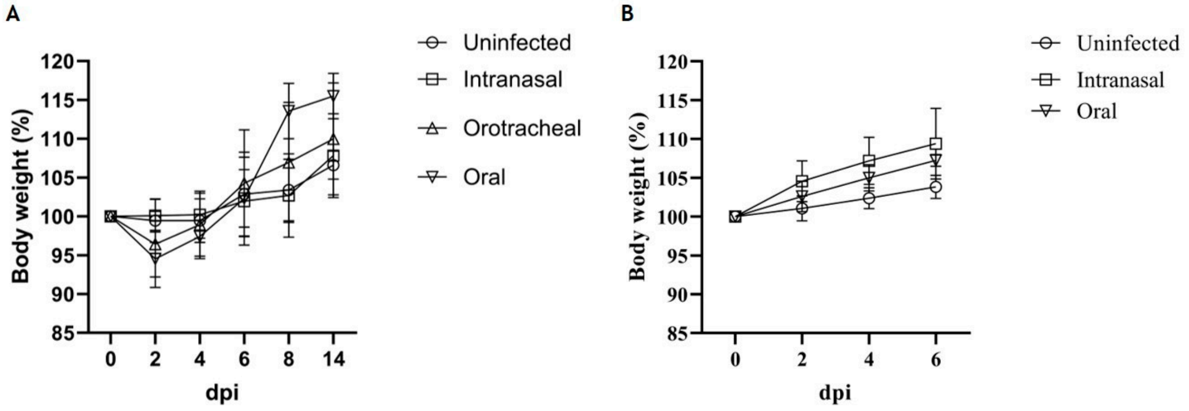

Supplementary Figure S1: Body weights of *R. aegyptiacus* bats after SARS-CoV-2 challenge using different inoculation routes (intranasal, orotracheal and oral). 6 bats per group were inoculated by the intranasal, orotracheal or oral route with a low SARS-CoV-2 dose of 10^4 $TCID_{50}$ and monitored for 14 days (A), or inoculated by the intranasal or oral route with a high dose of 10^6 $TCID_{50}$ and monitored for 6 days (B). 3 animals were kept as uninfected control animals in each study. Graphs show body weight changes (%) in relation to 0 dpi, bars indicate the mean and standard deviation.

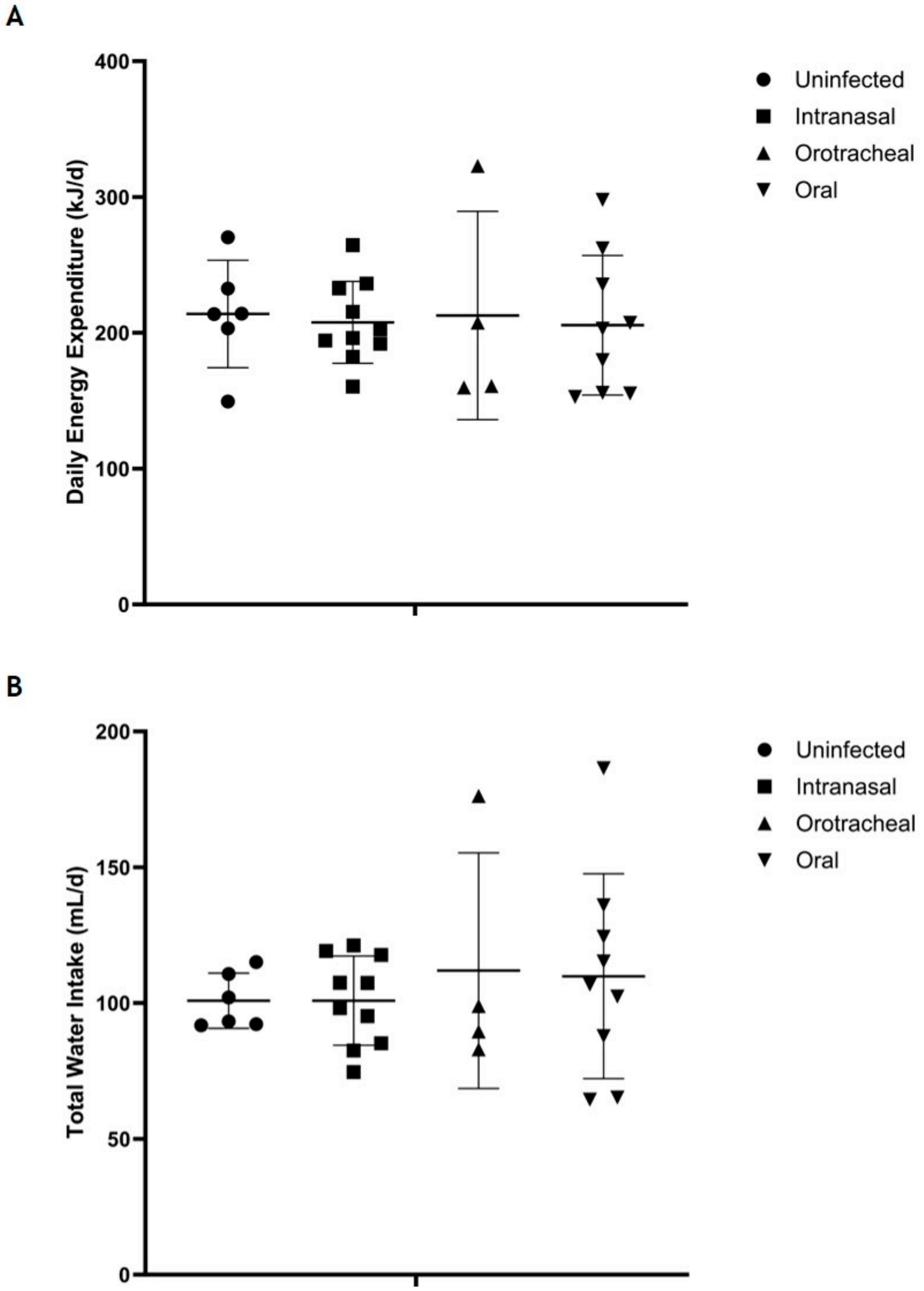

Supplementary Figure S2: Daily energy expenditure (A) and total water turnover (B) measured using the doubly labelled water method over two days in *R. aegyptiacus* either not infected or infected with SARS-CoV-2 via oral, intranasal or orotracheal inoculation.

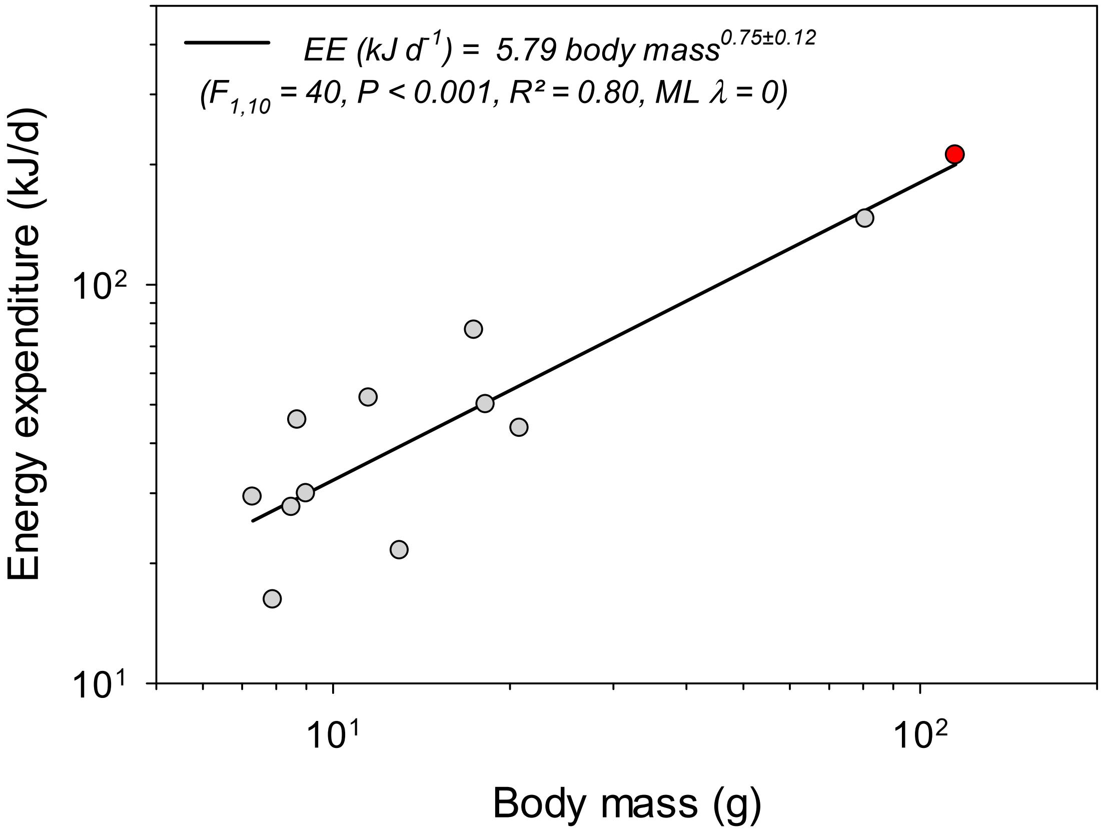

Supplementary Figure S3: Daily energy expenditure in bats. Relationship between energy expenditure (EE) and body mass in bats measured using the doubly labelled water method. Each grey data point represents a different bat species (n = 11, see Suppl. Table S2) and the red data point is the result of the uninfected bats from the present study on *R. aegyptiacus*.

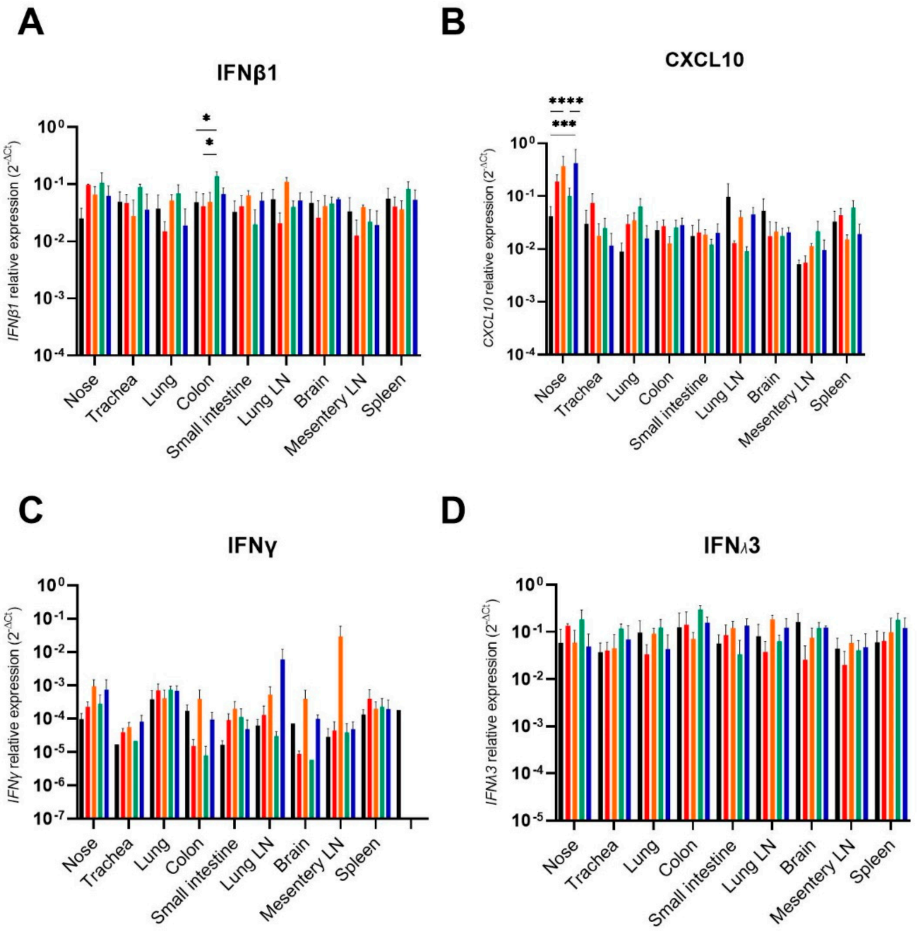

Supplementary Figure S4: Immune gene expression in intranasally or orally challenged *R. aegyptiacus* bats using the high SARS-CoV2 dose (10^6 $TCID_{50}$). Three Egyptian fruit bats / group were challenged intranasally with a high SARS-CoV-2 dose (10^6 $TCID_{50}$) and sacrificed at 2 (red) or 6 dpi (orange), or we challenged orally with the same dose and sacrificed at 2 (green) or 6 dpi (blue), while control animals were mock-infected with cell culture medium (black). The expression of *IFNβ1* (A), *CXCL10* (B), *IFNγ* (C) and *IFNλ3* (D) was analyzed. Data are shown as mean ± SEM; p-values were calculated using two-way ANOVA with Tukey's multiple comparison test. (*) $p < 0.05$, (**) $p < 0.01$, (***) $p < 0.001$, (****) $p < 0.0001$.

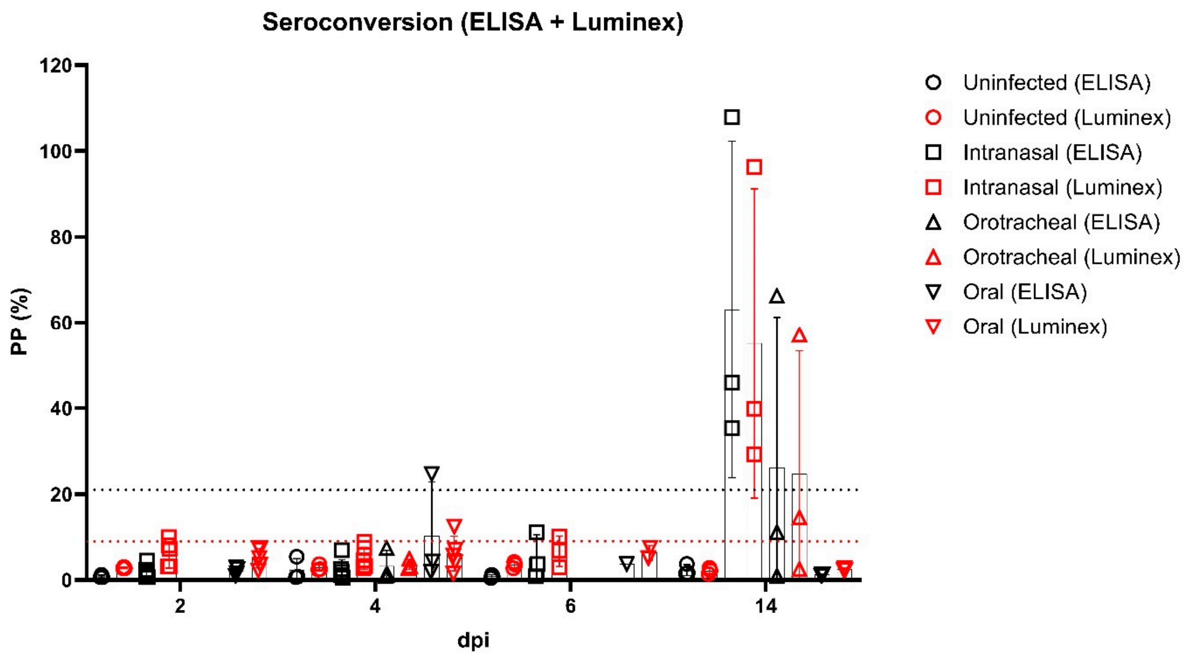

Supplementary Figure S5: SARS-CoV-2 microsphere-based assay (red) and ELISA (black) of bat sera collected at 2, 4, 6 and 14 dpi. Percent positive value (%) compared the positive control (PP). (2 dpi: uninfected n = 3, intranasal n = 6; oral n = 6; 4 dpi: uninfected n = 3, intranasal n = 6, orotracheal n = 3, oral n = 6; 6 dpi: uninfected n = 3, intranasal n = 3, oral n = 3; 14 dpi: uninfected n = 3, intranasal n = 3, orotracheal n = 3, oral n = 3). Intranasally and orotracheally challenged bats show seroconversion 14 dpi, while no serological response was detectable in orally challenged bats.

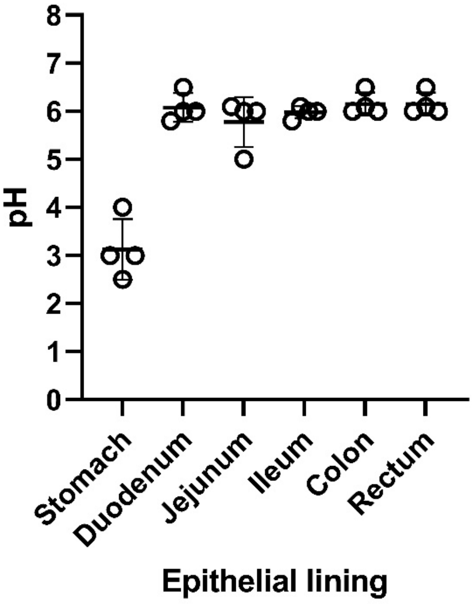

Supplementary Figure S6: pH at different parts of the *R. aegyptiacus* GIT. The epithelial lining of different sections of the GIT was accessed and pH measurements performed by swabbing the exposed epithelial lining with pH-measurement strips (n=4). The pH in the stomach is acidic with a mean value of 3, while the pH in all intestine specimens was close to neutral with a pH of 6.

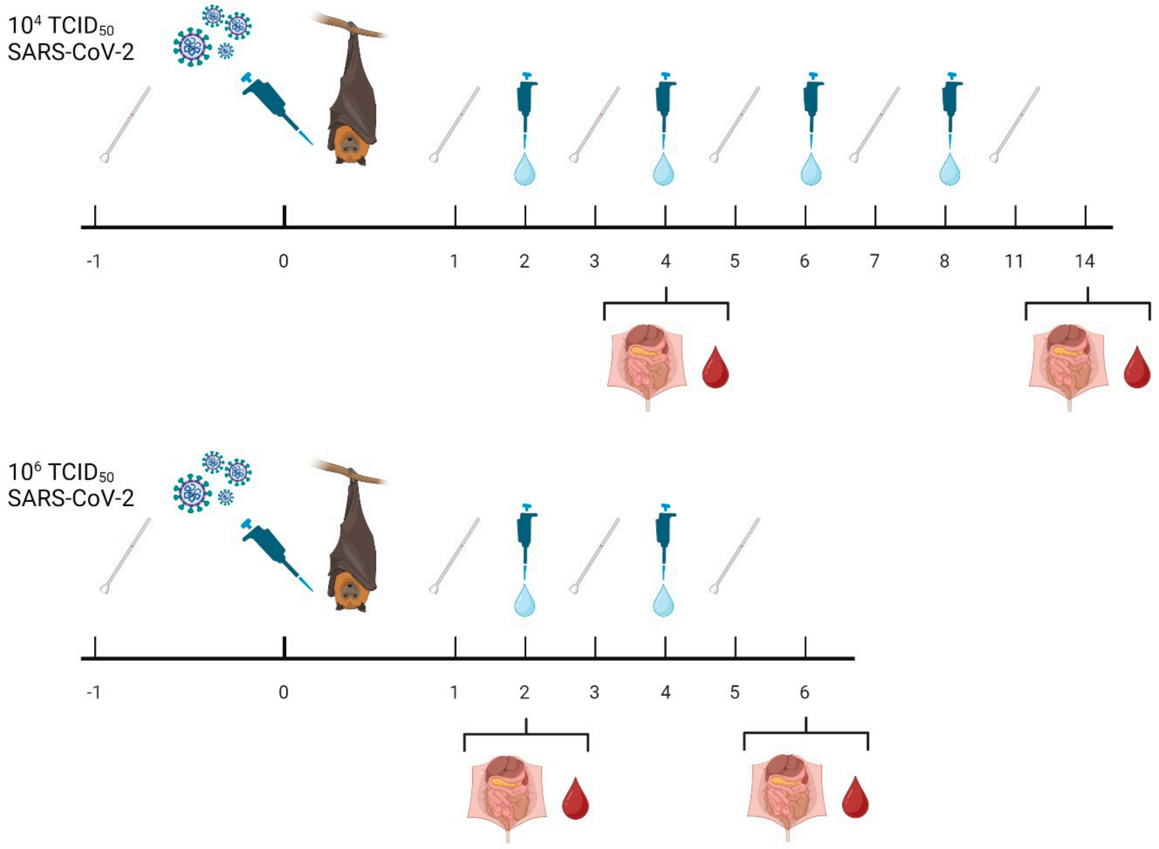

Supplementary Figure S7: Experimental outline. Graphical representation of the experimental outline for *R. aegyptiacus* bats infected with 10^4 $TCID_{50}$ (low dose) and 10^6 $TCID_{50}$ (high dose), including sample taking regime and necropsy time points.

Supplementary Table S1: Mean (day vs night) body temperature and locomotion activity measured in both studies in the different infection groups.

Infection dose	10^4 $TCID_{50}$		10^6 $TCID_{50}$	
Parameter	Body temperature (°C)	Locomotion activity (milli-g)	Body temperature (°C)	Locomotion activity (milli-g)
uninfected	36.2 ±0.85	11.49 ±10.09	36.73 ±1.04	15.42 ±12.16
intranasal	36.37 ±0.8	13.94 ±9	36.53 ±0.88	13.16 ±9.39
orotracheal	37.15 ±1.13	27.93 ±20.96	n.d.	n.d.
oral	37.03 ±1.57	26.27 ±21.92	36.72 ±1.02	20.09 ±15.22
uninfected	37.32 ±0.73	65.08 ±39.31	37.49 ±0.55	56.23 ±34.76
intranasal	37.6 ±0.64	54.78 ±29.74	37.5 ±0.52	41.58 ±20.69
orotracheal	37.08 ±0.86	42.38 ±28.81	n.d.	n.d.
oral	36.89 ±1.06	36.6 ±29.38	37.53 ±0.74	45.28 ±30.2

n.d. = not done; gray = night; white = day

Supplementary Table S2: Daily energy expenditure and body mass for 12 bat species

Phylogenetic tree in Newick format for 12 bat species for which daily energy expenditure has been measured using the doubly labelled water method:

((Rousettus_aegyptiacus:24.6,Syconycteris_australis:24.6):46.6,(Saccopteryx_bilineata:69.2,(((Macrotus_californicus:27.3,Phyllostomus_hastatus:27.3):0.5,((Glossophaga_commissarisi:19.4,Anoura_caudifera:19.4):7.3,Carollia_brevicauda:26.7):1.1):32.2,(((Pipistrellus_pipistrellus:26,Myotis_lucifugus:26):0,Plecotus_auritus:26):0,Eptesicus_fuscus:26):34):9.2):2);

**Species**	**BM (g)**	**FMR (kJ/d)**	**References**
Eptesicus_fuscus	20.8	43.6	(87)
Glossophaga_commissarisi	8.7	45.7	(86)
Macrotus_californicus	13.0	21.5	(87)
Myotis_lucifugus	9.01	29.9	(87)
Phyllostomus_hastatus	80.84	146.0	(87)
Pipistrellus_pipistrellus	7.3	29.3	(87)
Plecotus_auritus	8.5	27.6	(87)
Syconycteris_australis	17.4	76.9	(85)
Anoura_caudifera	11.49999999	51.9	(87)
Carollia_brevicauda	18.2	50.0	(86)
Saccopteryx_bilineata	7.9	16.2	(84)
Rousettus_aegyptiacus	115	211.0	Present study

Supplementary Table S3: Statistical analysis of RT-qPCR data

	Inoculation route	Sample	dpi	*p*-value
10^4 $TCID_{50}$/mL	Intranasal vs Oral	Oral swabs - gRNA	5	<0.05*
	Orotrachael vs Oral	Oral swabs - gRNA	5	<0.05*
	Intranasal vs Oral	Anal swabs - gRNA	7	<0.05*
	Orotrachael vs Oral	Anal swabs - gRNA	7	<0.05*
	Intranasal vs Oral	Nasal lavage - gRNA	2	<0.05*
	Orotrachael vs Oral	Nasal lavage - gRNA	2	<0.05*
	Intranasal vs Oral	Nasal lavage - gRNA	4	<0.05*
	Orotrachael vs Oral	Nasal lavage - gRNA	4	<0.05*
	Intranasal vs Oral	Nasal lavage - gRNA	6	<0.05*
	Orotrachael vs Oral	Nasal lavage - gRNA	6	<0.05*
	Intranasal vs Oral	Nasal lavage - gRNA	8	<0.05*
	Orotrachael vs Oral	Nasal lavage - gRNA	8	<0.05*
	Intranasal vs Oral	Lung - gRNA	4	<0.05*
	Orotrachael vs Oral	Lung - gRNA	4	NS
	Intranasal vs Oral	Brain - gRNA	4	<0.05*
	Orotrachael vs Oral	Brain - gRNA	4	NS

	Inoculation route	Sample	dpi	*p*-value
10^6 $TCID_{50}$/mL	Intranasal vs Oral	Anal swabs - gRNA	2	<0.05*
	Intranasal vs Oral	Anal swabs - gRNA	5	<0.05*
	Intranasal vs Oral	Nasal lavage - gRNA	2	<0.05*
	Intranasal vs Oral	Nasal lavage - gRNA	4	<0.05*
	Intranasal vs Oral	Nose - gRNA	2	<0.05*
	Intranasal vs Oral	Trachea - gRNA	2	<0.05*
	Intranasal vs Oral	Colon - gRNA	2	NS
	Intranasal vs Oral	Lung l.n. - gRNA	2	<0.05*
	Intranasal vs Oral	Brain - gRNA	2	<0.05*
	Intranasal vs Oral	Mesenteric l.n. - gRNA	2	<0.05*
	Intranasal vs Oral	Colon - gRNA	6	NS

No significant: NS, significant: *p*-value < 0.05*

Supplementary Table S4: ISH results

ID	Inoculation route	Pregnancy	Necropsy at dpi	ISH analysis and result
FH1	Uninfected control	yes	14	
FH2	Uninfected control	no	14	
FH3	Uninfected control	yes	14	
FH4	$1*10^4$ $TCID_{50}$ - Intranasal	yes	4	Intestine: negative
FH5	$1*10^4$ $TCID_{50}$ - Intranasal	no	4	
FH6	$1*10^4$ $TCID_{50}$ - Intranasal	no	4	lung, trachea, kidney, esophagus, stomach intestine: negative
FH7	$1*10^4$ $TCID_{50}$ - Intranasal	yes	14	
FH8	$1*10^4$ $TCID_{50}$ - Intranasal	no	14	
FH9	$1*10^4$ $TCID_{50}$ - Intranasal	no	14	
FH10	$1*10^4$ $TCID_{50}$ - Orotracheal	no	4	lung, trachea, esophagus, stomach, intestine: negative
FH11	$1*10^4$ $TCID_{50}$ - Orotracheal	no	4	
FH12	$1*1^4$ $TCID_{50}$ - Orotracheal	no	4	Intestine: negative
FH13	$1*10^4$ $TCID_{50}$ - Orotracheal	no	14	
FH14	$1*10^4$ $TCID_{50}$ - Orotracheal	no	14	
FH15	$1*10^4$ $TCID_{50}$ - Orotracheal	yes	14	
FH16	$1*10^4$ $TCID_{50}$ - Oral	yes	4	kidney, adrenal, brain: negative
FH17	$1*10^4$ $TCID_{50}$ - Oral	no	4	
FH18	$1*10^4$ $TCID_{50}$ - Oral	no	4	lung: negative
FH19	$1*10^4$ $TCID_{50}$ - Oral	no	14	
FH20	$1*10^4$ $TCID_{50}$ - Oral	no	14	
FH21	$1*10^4$ $TCID_{50}$ - Oral	no	14	
FH22	Uninfected control	no	6	
FH23	Uninfected control	no	6	

FH24	Uninfected control	no	6	
FH25	$1*10^6$ $TCID_{50}$ - Intranasal	no	2 dpi	cerebellum (neurons), cerebrum (menigeal cell, ventricle lining cell): **positive**<br>intestine, trachea, esophagus, lung, kidney, olfactory lobe of brain: negative<br>*chromogen precipitation intestinal lumen*
FH26	$1*10^6$ $TCID_{50}$ - Intranasal	no	2	Intestine: negative<br>*chromogen precipitation intestinal lumen*
FH27	$1*10^6$ $TCID_{50}$ - Intranasal	no	2	intestine, spleen, trachea, esophagus, stomach, liver, heart: negative<br>*chromogen precipitation intestinal lumen*
FH28	$1*10^6$ $TCID_{50}$ - Intranasal	no	6	Intestine: negative<br>*chromogen precipitation intestinal lumen*
FH29	$1*10^6$ $TCID_{50}$ - Intranasal	no	6	Intestine: negative<br>*chromogen precipitation intestinal lumen*
FH30	$1*10^6$ $TCID_{50}$ - Intranasal	no	6	Intestine: negative
FH31	$1*10^6$ $TCID_{50}$ - Oral	no	2	Intestine: negative<br>*chromogen precipitation intestinal lumen*

FH32	1*10^6 $TCID_{50}$ - Oral	no	2	
FH33	1*10^6 $TCID_{50}$ - Oral	no	2	Intestine: negative
FH34	1*10^6 $TCID_{50}$ - Oral	no	6	
FH35*	1*10^6 $TCID_{50}$ - Oral	no	6	brain, intestine, esophagus, lung, spleen, liver, thymus, heart, kidney, adrenal gland, trachea: negative *chromogen precipitation intestinal lumen*
FH36	1*10^6 $TCID_{50}$ - Oral	yes	6	

* FH 35: due to the unexpected virus distribution pattern observed in this animal indicating an accidental intranasal infection, this animal was excluded from the overall analysis.
